# Activation of the Complement System on Human Endothelial Cells by Urban Particulate Matter Triggers Inflammation-Related Protein Production

**DOI:** 10.3390/ijms22073336

**Published:** 2021-03-24

**Authors:** Myoung Su Choi, Hyungtaek Jeon, Seung-Min Yoo, Myung-Shin Lee

**Affiliations:** 1 Department of Otorhinolaryngology, Eulji University Medical Center, Eulji University School of Medicine, Daejeon 35233, Korea; mschoi@eulji.ac.kr; 2 Department of Microbiology and Immunology, Eulji University School of Medicine, Daejeon 34824, Korea; jhtjoy0405@gmail.com (H.J.); smyoo@eulji.ac.kr (S.-M.Y.)

**Keywords:** particulate matter, complement system, endothelial cell, inflammation

## Abstract

Exposure to particulate matter (PM) is becoming a major global health issue. The amount and time of exposure to PM are known to be closely associated with cardiovascular diseases. However, the mechanism through which PM affects the vascular system is still not clear. Endothelial cells line the interior surface of blood vessels and actively interact with plasma proteins, including the complement system. Unregulated complement activation caused by invaders, such as pollutants, may promote endothelial inflammation. In the present study, we sought to investigate whether urban PM (UPM) acts on the endothelial environment via the complement system. UPM-treated human endothelial cells with normal human serum showed the deposition of membrane attack complexes (MACs) on the cell surface via the alternative pathway of the complement system. Despite the formation of MACs, cell death was not observed, and cell proliferation was increased in UPM-mediated complement activation. Furthermore, complement activation on endothelial cells stimulated the production of inflammation-related proteins. Our results revealed that UPM could activate the complement system in human endothelial cells and that complement activation regulated inflammatory reaction in microenvironment. These findings provide clues with regard to the role of the complement system in pathophysiologic events of vascular disease elicited by air pollution.

## 1. Introduction

Particulate matter (PM) is a mixture of microscopic particles with complex compositions suspended in the atmosphere; it mostly arises from the combustion of fossil fuels or road dust. An increasing amount of evidence suggests that PM is related to adverse health effects [1,2,3]. Furthermore, the smaller the size of the particles, the more likely they are to escape the respiratory defense mechanism and invade deep into the respiratory tract, which can directly affect the cells lining the alveolar wall [4]. PM is known to be associated not only with respiratory diseases [5] but also with cardiovascular and immunological disorders [6,7]. PM with a diameter of less than 2.5 μm can enter systemic circulation through gaps formed between alveolar epithelial cells [8]. PM in air pollution disrupts the endothelium and induces an inflammatory reaction, which is correlated to cardiovascular diseases [6,9,10]. However, the pathophysiologic mechanisms underlying the association between PM and cardiovascular disease are not fully understood.

The components of PM include organic carbon, elemental carbon, nitrate, sulfate, and trace metals [11]. Among them, quinones or organic compounds can generate superoxide anion, a type of reactive oxygen species (ROS) [12,13]. If the balance between the level of ROS generated by the PM and that of antioxidants armed against it is disturbed, cells or tissues will be exposed to oxidative stress, which is a significant factor in endothelial cell dysfunction, thrombosis, and inflammation [14,15].

The complement system has a central role in the innate immune response. Generally, it has been known to defend against invading pathogens; however, several studies have shown that complement activation plays a vital role in the pathophysiology of atherosclerosis and thrombosis [16,17]. Some experimental studies using the ischemia-reperfusion model indicated that complement activation is a significant factor contributing to tissue injury [18]. Inhibition of the lectin pathway of the complement system in myocardial ischemia inhibited complement activation and preserved the myocardium [19]. Complement C4 (-/-) and immunoglobulin M (-/-) knockout mice exhibited a decreased deposition of C3 and were protected from ischemic reperfusion injury [20]. These studies suggest that endothelial inflammation induced by unregulated complement activation is closely related to vascular injuries. Therefore, it is necessary to investigate the underlying mechanisms through which endothelial cells lose control of the complement system. A study investigating the serum levels of complement components in children living in areas exposed to different air pollution levels revealed that C3c and C4 were high in the serum of children who lived in Osaka, which has heavily polluted air [21]. Another study reported that serum levels of the immunoglobulin (Ig) A, IgM, and C3c were positively related to the degree of air pollution [22]. Tobacco smoke extracts also have been known to induce C3a and C5a production [23]. In summary, air pollution seems to be closely related to complement activation, but little is known of the mechanisms that initiate and regulate the complement system in response to PM.

To analyze the activation of the complement system in eukaryotic cells, several researchers used C3 protein deposition because C3b deposition on the cell surface is a common event in all complement activation [24,25], including classical and alternative pathways. However, since C3b is not a final product but rather an intermediate product of the complement activation process, the membrane attack complex C5b-9 would be a more reliable marker for complement activation. In our previous study, we established an enzyme-linked immunosorbent assay (ELISA)-based method to quantify C5b-9 deposition on the surfaces of eukaryotic cells [26], and this method was applied to determine the activation of the complement system in urban PM (UPM)-exposed human endothelial cells.

In the present study, we investigated whether urban PM can activate the complement system in human endothelial cells, along with the underlying mechanisms involved. To the best of our knowledge, this is the first study to demonstrate the direct association between UPM and complement activation in an experimental cell culture model. Our results might be helpful in clarifying the role of UPM-mediated complement activation in the various pathophysiological effects derived from air pollutants.

## 2. Results

### 2.1. Effects of UPM on Human Endothelial Cell Viability and Complement Activation

**Figure 1:**
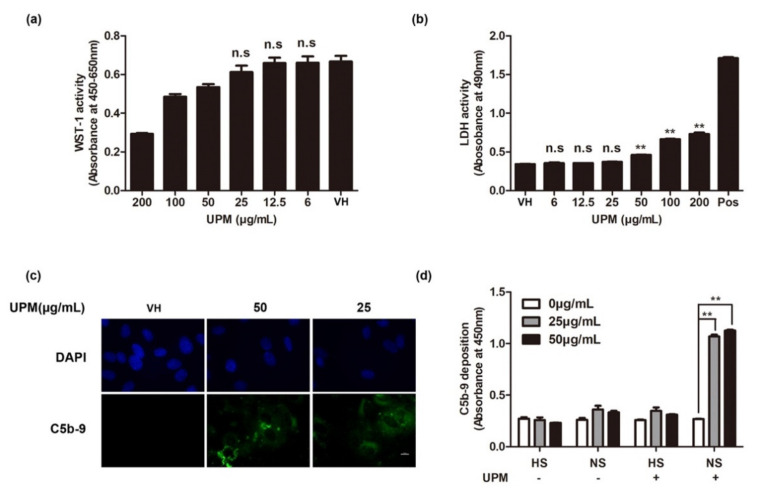
Effects of urban particulate matter (UPM) on human umbilical vein endothelial cell (HUVEC) viability and complement activation. (**a**) The viability of UPM-treated HUVECs. HUVECs were exposed to UPM for 24 h (200 μg/mL to 0 μg/mL). Cell viability was analyzed using the WST-1 assay. Data are represented as mean ± standard deviation (SD) of the mean (*n* = 5). One-way ANOVA with multiple comparisons. The mean of each column with the mean of the VH column. n.s = not significant. ** *p* < 0.01. VH: vehicle control (5% methanol). (**b**) Cytotoxicity of UPM in HUVECs. Lactate dehydrogenase (LDH) activities were measured in HUVECs exposed to UPM for 24 h. Data are represented as mean ± SD, *n* = 5. One-way ANOVA with multiple comparisons. n.s = not significant. ** *p* < 0.01. (**c**) Detection of C5b-9 depositions on UPM-treated HUVECs by immunofluorescence assay (IFA). HUVECs were treated with UPM for 24 h (0 μg/mL, 25 μg/mL, and 50 μg/mL), followed by treatment with 10% normal human serum (NS) for another 30 min. The C5b-9 deposition was detected by IFA with an anti-C5b-9 antibody. Nuclei were stained with DAPI (blue), C5b-9: deposition of C5b-9 (green). Scale bar: 10 μm. (**d**) Analysis of C5b-9 depositions by cell-based enzyme-linked immunosorbent assay (ELISA). UPM-exposed HUVECs were treated with heat-inactivated human serum (HS) or NS for 30 min, followed by analysis of C5b-9 depositions using cell-based ELISA. Results are shown as mean ± SD, *n* = 5, One-way ANOVA with multiple comparisons. ** *p* < 0.01.

To determine the cytotoxicity of UPM, human umbilical vein endothelial cells (HUVECs) were treated with various concentrations of UPM (0, 6, 12.5, 25, 50, 100, or 200 μg/mL) for 48 h. A significant decrease in cell viability and an increase in cell death were observed at 48 h after treatment with 50 to 200 μg/mL UPM compared to the untreated control (Figure 1a,b). To investigate whether exposure to UPM can result in complement activation, we examined the deposition of membrane attack complexes (MACs) or C5b-9 on untreated and UPM-treated HUVECs at 48 h following incubation of the cells with normal human serum (NS) for 1 h. The deposition of MACs was observed on UPM-treated cells but not on untreated cells (Figure 1c). As the deposition of MACs was induced at a concentration that did not induce cell death (25 μg/mL), complement activation would not have been mediated by cell death. To quantify the deposition of C5b-9 on cells, a cell-based ELISA technique was applied [26]. We observed approximately fourfold higher signals for C5b-9 depositions on UPM-treated cells than on untreated cells (Figure 1d). Heated human serum (HS) was used as a negative control, and C5b-9 deposition was not observed on HS-treated cells even after UPM treatment. In summary, our findings indicate that UPM results in complement activation in HUVECs even at a concentration that does not induce cell death.

### 2.2. UPM Activates the Complement System in HUVECs via the Alternative Pathway

**Figure 2:**
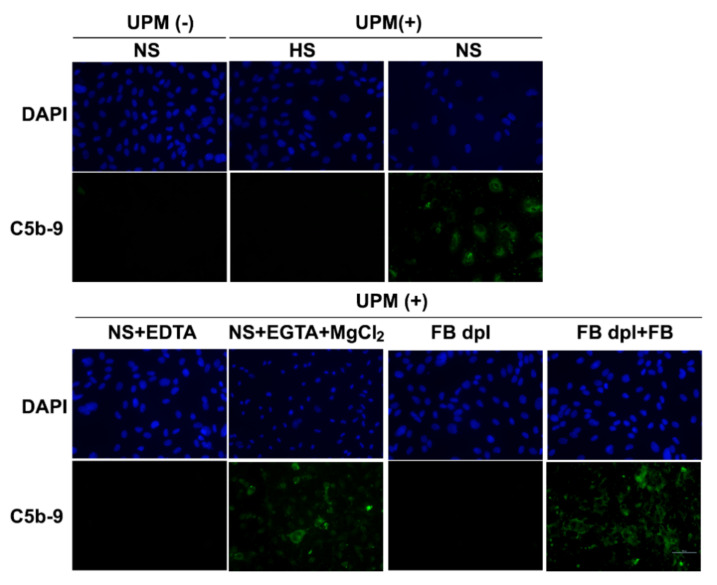
Activation of the alternative pathway of the complement system in UPM-treated HUVECs. Untreated or UPM-treated HUVECs were incubated with heated human serum or normal human serum. Then, 20 mM EDTA and 10 mM EGTA with 20 mM MgCl_2_ were used to inhibit the entire pathway and the classical pathway of the complement system, respectively. Factor B-depleted serum (FB Dpl sera), or Factor B (FB) was used to investigate whether the alternative pathway was activated in UPM-treated HUVECs. Nuclei were stained with DAPI (blue), C5b-9: deposition of C5b-9 (green). Scale bar: 50 μm.

To identify the pathway of the complement system activated by UPM, UPM stimulated cells were incubated with NS containing 10 mM ethylenediaminetetraacetic acid (EDTA) or 10 mM ethylene glycol-bis(2-aminoethylether)-N,N,N′,N′-tetraacetic acid (EGTA) with 2 mM MgCl_2_. EDTA inhibits the activation of all the complement pathways, while EGTA with 2 mM MgCl_2_ specifically inhibits the antibody-dependent classical complement pathway. EDTA- and UPM-treated cells no longer showed C5b-9 deposition, whereas cells treated with EGTA together with MgCl_2_ showed C5b-9 deposition (Figure 2). These results indicated that C5b-9 deposition on UPM-treated cells was not mediated by the classical complement pathway. To determine whether the activation of the alternative complement pathway was involved in C5b-9 deposition during UPM treatment, we treated the cells with factor B-depleted serum. Factor B is an essential component for initiating the alternative complement pathway. Depletion of factor B failed to induce C5b-9 deposition in UPM-mediated complement activation; however, addition of factor B to the factor B-depleted human serum rescued C5b-9 deposition (Figure 2). The above results indicated that the alternative complement pathway was activated during UPM stimulation of HUVECs.

### 2.3. UPM-Mediated Complement Activation of HUVECs Is Associated with ROS But Not Complement Regulatory Proteins

**Figure 3:**
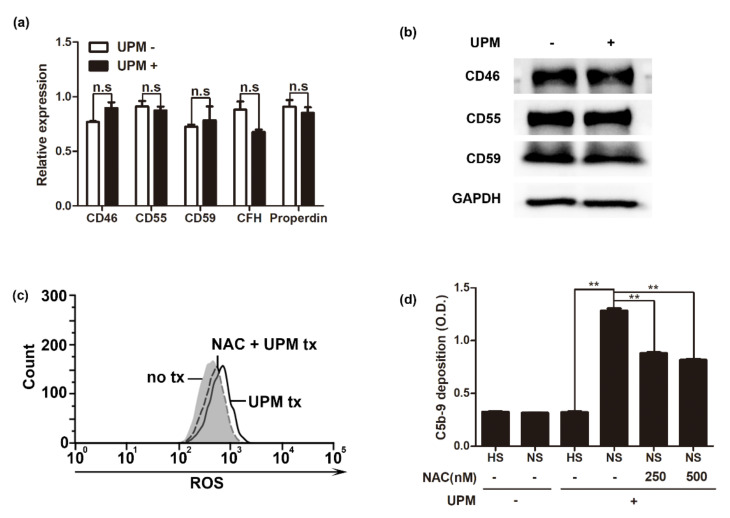
Complement activation in UPM-treated HUVECs is mediated by reactive oxygen species (ROS) production and not complement regulatory proteins (**a**) The mRNA expression of CD46, CD55, CD59, and factor H in UPM-treated HUVECs. Relative expression of each indicated protein was analyzed by reverse transcription (RT)-quantitative PCR. Results are shown as mean ± SD, *n* = 3, Student’s *t*-test. n.s: not significant. (**b**) Western blot analysis for CD46, CD55, and CD59 protein expression in UPM-treated HUVECs. (**c**) Cellular ROS production in UPM-treated HUVECs. HUVECs were labeled with CellROX Green reagent and analyzed by flow cytometry. N-acetylcysteine (NAC) was added for 3 h before UPM treatment. Gray: untreated HUVECs (control). Black dot line: NAC-treated HUVECs with UPM (NAC + UPM), black line: HUVECs with UPM (UPM). (**d**) Analysis of C5b-9 depositions on NAC-treated HUVECs with/without UPM using a cell-based enzyme-linked immunosorbent assay. Results are shown as mean ± SD, *n* = 5, Student’s *t*-test (HS vs. NS) or one-way ANOVA with multiple comparisons. ** *p* < 0.01.

In the alternative pathway, complement regulatory proteins have a crucial role in complement system activation. Expression of complement regulatory proteins such as CD46, CD55, and CD59 on the cell surface inhibits complement activation [27]. To investigate whether UPM regulates the expression of the complement regulatory proteins in human endothelial cells, the expression profiles of CD46, CD55, CD59, and factor H were analyzed in HUVECs following UPM treatment. We found that UPM did not affect the mRNA expression of CD46, CD55, CD59, and Factor H (Figure 3a). The protein expression profiles of CD46, CD55, and CD59 were not affected by UPM treatment (Figure 3b), suggesting that complement activation following UPM treatment of HUVECs was not caused by the suppression of these proteins. Since UPM is known to induce oxidative stress [28], we investigated the relationship between ROS production following UPM treatment and complement activation. After the HUVECs were treated with UPM, ROS production was measured by flow cytometry with CellROX reagents (Figure 3c). UPM-treated HUVECs showed higher ROS production, and N-acetylcysteine (NAC) suppressed UPM-mediated ROS production. Suppression of ROS by NAC attenuated complement activation (Figure 3d), indicating that ROS are associated with complement activation following UPM treatment.

### 2.4. Cell Viability in Complement-Activated HUVECs Following UPM Treatment

**Figure 4:**
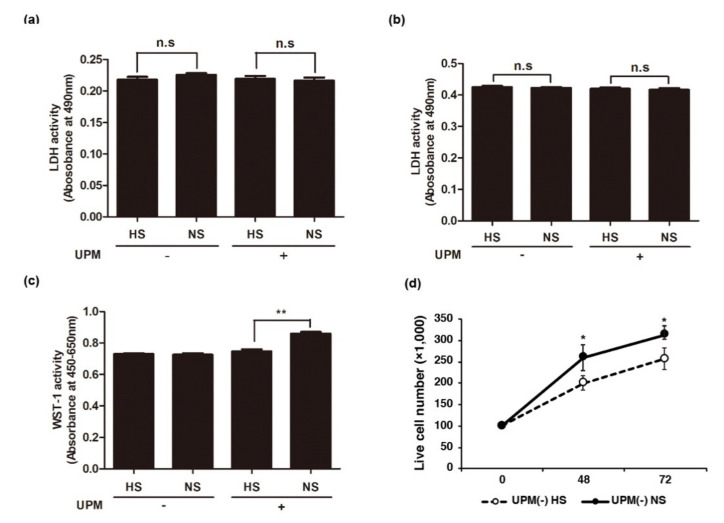
Cell death and proliferation of UPM-treated HUVECs with human serum. (**a**) LDH assay in UPM-treated HUVECs incubated with heated human serum (HS) or normal human serum (NS) for 1 h. Untreated (UPM-) or UPM-treated HUVECs (UPM+) incubated with HS or NS for 1 h. LDH activities were measured in the supernatant of the cultured cells. Results are shown as mean ± SD, *n* = 5, Two-way ANOVA with multiple comparisons. n.s: not significant. (**b**) LDH assay in UPM-treated HUVECs incubated with HS or NS for 48 h. After complement activation for 1 h, culture medium was replaced with fresh medium to remove UPM and human serum. Cells were incubated for 48 h, and cell death was measured by LDH assay. Results are shown as mean ± SD, *n* = 5, Two-way ANOVA with multiple comparisons. n.s: not significant. (**c**) Proliferation of HUVECs with UPM-mediated complement activation. Heated or normal human serum was applied to UPM-treated HUVECs for 1 h, after which the media were replaced to remove UPM and human serum. After incubation with fresh media for 48 h, cell proliferation was measured using the WST-1 assay. Results are shown as mean ± SD, *n* = 5, Two-way ANOVA with multiple comparisons. ** *p* < 0.01. (**d**) Analysis of live cell numbers of HUVECs with UPM-mediated complement activation. Trypan blue exclusion assay was applied to cell count at 48 h and 72 h after exposure of UPM. Student-*t* test. Results are shown as mean ± SD, *n* = 3, * *p* < 0.05.

Complement activation can lead to cell lysis by forming MACs on the cell surface [29,30]. Therefore, we investigated whether cell viability was affected by complement activation in HUVECs. As shown in Figure 4a, we observed a similar level of lactate dehydrogenase (LDH) activity in UPM-exposed cells treated with NS or HS for 1 h (Figure 4a), indicating that complement activation does not induce cell death in UPM-exposed endothelial cells. This result is not surprising, as previous studies have demonstrated that complement activation through the alternative pathway does not induce cell death [25,31]. To investigate the effect of complement activation for a longer time period, the culture medium was replaced with fresh medium without UPM and NS following complement activation on treatment with UPM and NS for 1 h. Then, we cultured the cells for 48 h, and cell death and viability were measured, respectively, using LDH and WST-1 assays. A similar level of LDH activity was observed in NS- and HS-treated cells (Figure 4b). However, the viability of PM-exposed HUVECs following complement activation by NS was significantly higher than that of HS-treated cells (Figure 4c). These results suggested that complement activation may stimulate proliferation of UPM-treated endothelial cells.

### 2.5. UPM-Mediated Complement Activation Induces the Phosphorylation of STAT3

**Figure 5:**
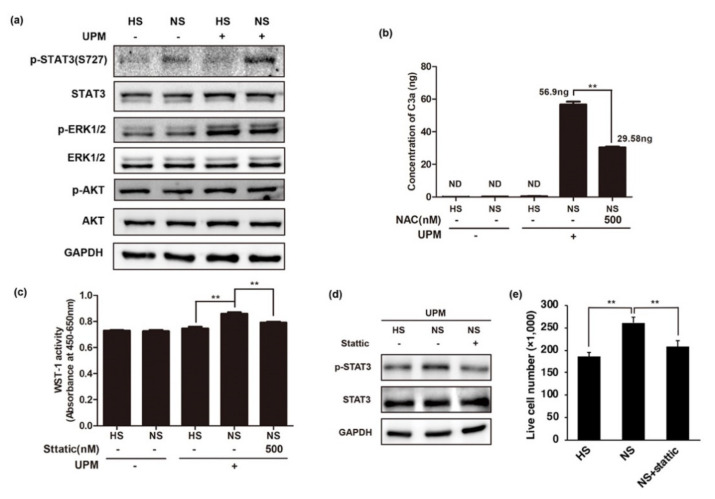
Complement activation provides survival benefit in UPM-treated HUVECs by inducing STAT3 phosphorylation. (**a**) Western blot for complement activation-related signaling proteins. UPM-treated HUVECs were incubated with heated human serum or normal human serum for 48 h. Each indicated protein was analyzed with cell lysate using Western blotting. (**b**) Analysis of C3a in the culture supernatant from UPM-treated HUVECs with human serum. The culture supernatant from UPM-treated HUVECs with HS or NS was collected, and C3a was analyzed by ELISA. One hour before the serum was added, NAC was applied to UPM-treated HUVECs. Results are shown as mean ± SD, *n* = 3, Two-way ANOVA and multiple comparisons. ** *p* < 0.01. (**c**) STAT3 inhibitor, stattic, attenuated the complement activation-mediated survival benefit of UPM-treated HUVECs. The same number of cells were seeded onto a 96-well culture plate. HUVECs were treated with UPM for 24 h, followed by the indicated HS or NS for 1 h. Cells were treated with stattic 2 h before human serum treatment. HS or NS was applied for 1 h, and the culture medium was changed into fresh medium. After incubation for 48 h, cell viability was measured using the WST-1 assay. Results are shown as mean ± SD, *n* = 5, two-way ANOVA and multiple comparisons. ** *p* < 0.01. (**d**) Western blot for *p*-STAT with a specific inhibitor, stattic (500 nM). (**e**) Analysis of live cell numbers of HUVECs with UPM-mediated complement activation. Stattic was applied at 500 nM. Trypan blue exclusion assay was applied to cell count at 48 h after exposure of UPM. One-way ANOVA. Results are shown as mean ± SD, *n* = 3, ** *p* < 0.01.

C3a/C5a fragments, also known as anaphylatoxins, are produced during the complement activation process by cleavage of C3 or C5 proteins. C3a/C5a can stimulate multiple signaling pathways through the interaction with their receptor on the cell surface [32]. Interaction between C3a and C3a receptor activates STAT3 and causes an increase in Wnt2b and SOX-2 expression in a serine protease MAPK-dependent fashion, as shown in a model of retinal regeneration [33]. Furthermore, C5a receptor activation contributes to the maintenance of pluripotency of OCT-4 positive human induced pluripotent stem cells through ERK1/2 activation or AKT1/2 activation [34]. HUVECs constitutively express both these receptors on their cell surface [35]. To investigate whether cell proliferation is caused by C3a or C5a, we analyzed the anaphylatoxins receptor-related signaling pathways, including AKT, ERK, and STAT3 (Figure 5a). Our results indicated that AKT and ERK pathways are not activated in UPM-treated HUVECs with NS. While the phosphorylation of ERK was increased in UPM-treated HUVECs compared to that in untreated cells, complement activation did not affect ERK signaling. These results indicate that the phosphorylation of ERK may be induced by UPM, but not complement activation. The phosphorylation of AKT was not changed by either UPM or complement activation. Intriguingly, the phosphorylation of STAT3 was increased in UPM-treated HUVECs incubated with NS compared to that with HS, suggesting that complement activation induces the phosphorylation of STAT3. C3a expression was analyzed by ELISA with the supernatant from cells. As expected, while C3a was not detected in the supernatant from cells without complement activation, UPM-exposed HUVECs incubated with NS showed C3a expression. Additionally, suppression of ROS in these cells by NAC showed less amount of C3a production, suggesting that C3a protein level could be correlated with complement activation (Figure 5b). We investigated whether the phosphorylation of STAT3 is associated with complement system-mediated cell proliferation (Figure 4c); cell proliferation was analyzed in the presence of an inhibitor of STAT3 with the same conditions (Figure 5c). We found that the viability of UPM- and NS-treated HUVECs was significantly attenuated by the STAT3 inhibitor.

### 2.6. UPM-Mediated Complement Activation Stimulates the Production of Inflammatory Cytokines

**Figure 6:**
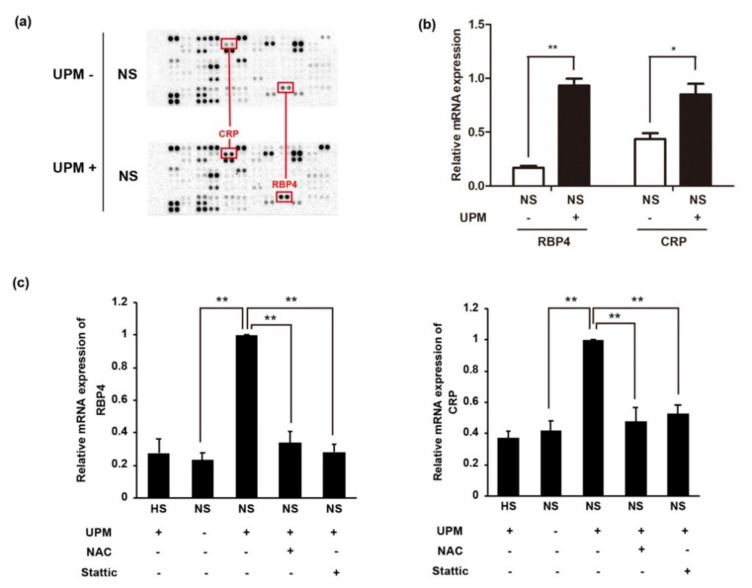
Complement activation induces the expression of inflammation-related proteins in UPM-treated HUVECs. Normal human serum was added to UPM- or vehicle (5% methanol)-treated HUVECs for 1 h, followed by replacement of the medium and incubation for 24 h. RNA and culture supernatant were collected to analyze mRNA and protein expression, respectively. (**a**) Antibody array with the supernatant from UPM-treated HUVECs using a Proteome Profiler Human XL Cytokine Array Kit from R&D Systems. The red box indicates the upregulated proteins in the supernatant from UPM-treated cells (UPM+) compared to the vehicle-treated cells (UPM-). CRP: C-reactive protein, RBP4: retinol binding protein 4. (**b**) The mRNA expression of complement activation-related proteins (RBP4 and CRP) in UPM-treated HUVECs with human serum. The mRNA expression was analyzed by RT-qPCR. Results are shown as mean ± SD, *n* = 3, Student’s *t*-test. * *p* < 0.05, ** *p* < 0.01. (**c**) Suppression of the mRNA expression of complement activation-related proteins (RBP4 and CRP) in UPM-treated HUVECs incubated with human serum by NAC or STAT3 inhibitor, stattic. NAC was added for 3 h before UPM treatment. Stattic was added to HUVECs 2 h before human serum treatment. Results are shown as mean ± SD, *n* = 3, one-way ANOVA and multiple comparisons. * *p* < 0.05, ** *p* < 0.01.

Anaphylatoxins generated through complement activation can lead to the secretion of cytokines and chemokines through the activation signals [36]. Since STAT3 was activated in UPM- and NS-treated HUVECs, we wondered if cytokine production is stimulated in these cells. To determine whether cytokine productions are enhanced by UPM-mediated complement activation, the culture supernatant from UPM-treated HUVECs with HS/NS was applied to a Proteome Profiler Human XL Cytokine Array Kit from R&D Systems (Figure 6a). We found that two inflammation-related proteins, including C-reactive protein (CRP) and retinol binding protein 4 (RBP4), were upregulated in UPM-treated cells with NS compared to those without UPM (Figure 6a). Their mRNA expressions were also consistent with the result of the protein array (Figure 6b). The inhibition of ROS production by NAC suppressed the expression of RBP4 and CRP in UPM-exposed HUVECs with NS, which would be the result of suppressing complement activation (Figure 6c). Finally, to investigate if STAT3 is associated with the production of these proteins by complement activation, UPM-exposed HUVECs with NS were subjected to STAT3 inhibitor treatment (Figure 6c). Interestingly, the production of both RBP4 and CRP was significantly suppressed by STAT3 inhibition, suggesting that the STAT3 pathway may participate in the production of these inflammation-related proteins in UPM-treated HUVECs with complement activation.

## 3. Discussion

We found that the exposure of HUVECs to UPM increased the production of ROS and activated the complement system via the alternative pathway on addition of NS. Furthermore, treatment with the antioxidant NAC significantly reduced C5b-9 deposition on HUVECs, which suggested that oxidative stress was associated with the complement activation. Several previous studies showed that oxidative stress can activate the complement system [37,38]. However, the complement activation observed in our study is different from that observed in previous studies in that the activation of the complement system was induced by the alternative pathway and not the classical pathway. Since the suppression of ROS production partially attenuated the complement activation in UPM-induced HUVECs, the complement activation by UPM may have been mediated by other factors. A recent study indicated that oxidative stress increased complement proteins and receptors in retinal pigment epithelial cells [39]. Increased complement proteins by ROS may be associated with enhanced complement activation in UPM-exposed HUVECs.

PM is deposited on the lung parenchyma through inhalation, of which ultrafine PM with a size smaller than 100 nm (0.1 µm) is known to be capable of translocation to the capillary. To the best of our knowledge, there are no methods available for measuring the level of PM in blood vessels due to a lack of measuring tools and the sparse concentration of PM in blood. Therefore, we determined PM concentrations in this study using the PM concentrations applied in previous studies targeting vascular endothelial cells [40].

The final assembly of MACs is classically known for cell lysis by making pores in the plasma membrane of targeted cells or pathogens. However, nucleated cells can avoid and protect themselves from lysis by the expression of inhibitor proteins that interfere with MAC assembly or by clearing of MACs via endocytosis or shedding. Furthermore, sublytic MACs may elicit intracellular signaling and various inflammatory responses [41]. In our previous study, complement-activated supernatant from NS-treated osteosarcoma cell lines demonstrated enhancement of angiogenesis in HUVECs through increased growth factor production, such as vascular endothelial growth factor-A and fibroblast growth factor-1 [42]. A murine model of laser-induced choroidal neovascularization confirmed the deposition of MACs and C3b in the neovascular complex. However, choroidal neovascularization was not developed in C3 knockout mice [43].

Anaphylatoxins, including C3a and C5a, are well-known for mediating histamine release from mast cells, recruitment of blood myeloid cells to sites of inflammation, and contraction of the smooth muscle [44,45]. C3a and C5a signal their biological function by binding to their G protein-coupled receptors, the C3a receptor (C3aR) and C5a receptor (C5aR1), respectively. Constitutively, expression of C3aR and C5aR1 was demonstrated in cultured HUVECs by flow cytometric analysis [46]. Stimulation with IL-1β or IFN-γ caused an elevation in C3aR on the surface of HUVECs [35]. In an embryonic chick retina regeneration study, the interaction of C3a with C3aR induced retina regeneration via the activation of signal transducer and activator of transcription 3 (STAT3) [33]. In our study, it was confirmed that the expression of p-ERK1/2 or p-Akt was not changed, but p-STAT3 was increased when the complement system was activated. Moreover, C3a adopts STAT3 as a downstream signaling pathway [32]. Our results reveal that STAT3 could be associated with the enhanced proliferation of UPM-exposed HUVECs. Some previous studies showed that complement activation could provide a survival signal to various eukaryotic cells by anaphylatoxin via its receptor interaction or sublytic terminal complement components [42,47,48].

Complement protein C3a/C5a can stimulate the transcription of inflammatory cytokines in monocytes and macrophages [49,50]. Moreover, sublytic C5b-9 has been reported to induce the production of various cytokines [51,52]. Our results demonstrated that the complement activation on HUVECs following exposure to UPM enhanced the secretion of inflammation-related proteins, CRP and RBP4. CRP belongs to the pentraxin family of calcium dependent ligand-binding plasma proteins and is known as one of the secreted pattern recognition receptors [53]. CRP provides the first line of defense against pathogens and binds to dead or damaged cells. CRP is not only a biomarker of inflammation, but it is also a direct participant in atherogenesis [54]. Elevation of serum RBP4 is known to be linked with various cardiovascular diseases [55,56,57]. A previous study showed that RBP4 induces inflammation in HUVECs by stimulating expression of proinflammatory molecules [58]. Our results suggest the possibility of STAT3 being involved in production of CRP and RPB4 by HUVECs via the combination effect of UPM and complement activation. Further studies are needed to decipher the exact underlying mechanism and elucidate the role of the STAT3 pathway in the production of these inflammation-related proteins by HUVECs following UPM exposure and subsequent complement activation. The current study was conducted only in vitro, and its findings should be confirmed in an in vivo model in further study.

## 4. Materials and Methods

### 4.1. Reagents and Antibodies

The standard reference material for urban particulate matter (NIST^®^ SRM^®^1648a, UPM) was purchased from the National Institute of Standards and Technology (NIST, Gaithersburg, MD, USA). NAC was purchased from Merck (Darmstadt, Germany), and distilled water (DW) was used for vehicle control for NAC. CellROX™ Green Reagent for oxidative stress detection was purchased from Thermo Scientific (Waltham, MA, USA). EDTA, EGTA, and magnesium chloride (MgCl_2_) were purchased from Merck. STAT3 inhibitor, stattic, was purchased from Cell Signaling Technology (#97598, Danvers, MA, USA), and dimethyl sulfoxide (DMSO) was used as a vehicle for stattic. Pooled complement human serum was purchased from Innovative Research, Inc. (Novi, MI, USA) and used as normal human serum in all experiments. Heat inactivation was performed with this serum at 56 °C for 30 min. Factor B-depleted human serum and purified factor B were purchased from Innovative Research, Inc.

Antibodies against cyclooxygenase phospho-ERK, ERK, CD46, CD55, and CD59 were purchased from Santa Cruz Biotechnology (Santa Cruz, CA, USA). Antibody against glyceraldehyde 3-phosphate dehydrogenase (GAPDH) was purchased from Cusabio Technology (Houston, TX, USA). Antibodies against STAT3 and phospho-STAT3 were purchased from Cell Signaling Technology (Beverly, MA, USA). Antibodies against AKT and phospho-AKT were purchased from Bioss (Woburn, MA, USA). Antibody against C3 was purchased from Thermo Scientific (Waltham). The mRNA primers were synthesized by GenoTech (Daejeon, South Korea). The reagents used for real-time PCR and cDNA synthesis kits were purchased from Takara Bio (Shiga, Japan). The reagents used for Western blotting were obtained from Bio-Rad (Hercules, CA, USA).

### 4.2. Cell Culture

HUVECs were purchased from Lonza (Allendale, NJ, USA) and cultured in endothelial cell growth medium-2 (EGM-2) (Lonza) at 37 °C in a humidified atmosphere of 5% CO_2_.

### 4.3. Exposure of HUVECs to UPM and Other Inhibitors

For preparation of UPM stock solution, UPM was suspended in 5% methanol (500 mg/mL) and sonicated using the Branson Sonifier cell disruptor (Danbury, CT, USA) for 30 s. Unless otherwise noted, HUVECs were exposed to UPM at different concentrations for 24 h; 5% methanol was used for no-treatment control of UPM. HUVECs were treated with inhibitors NAC (500 nM) or stattic (500 nM) for 3 h before UPM stimulation. NS or heat-inactivated human serum was mixed in the culture medium and treated with the cells at a concentration of 10% for 1 h.

### 4.4. Reverse Transcription Quantitative PCR

Total RNA from cells was isolated using a NucleoSpin RNA II kit as per the manufacturer’s instructions (Macherey-Nagel Inc., Bethlehem, PA, USA). Total RNA was reverse transcribed to obtain the cDNA using the PrimeScript™ RT Master Mix (Takara). Quantitative PCR was performed using the SYBR^®^ FAST qPCR mix (Takara) on a real-time PCR system (Bio-Rad). The primer sequences are shown in Table 1. The relative level of mRNA expression of each target gene is shown as a 2−△△Ct value. GAPDH was used as an internal control.

### 4.5. Western Blotting

The total protein of cells in different groups was obtained using RIPA buffer containing phenylmethanesulfonyl fluoride (UPMSF, Roche, Basel, Switzerland), protease inhibitor cocktail (Roche), and phosphatase inhibitors. The protein concentration was measured using the BCA Protein Assay kit (Thermo Scientific). An identical amount of lysates (20–30 µg/well) was subjected to separation using SDS-PAGE and the protein bands were then transferred onto nitrocellulose membranes. Subsequently, the membranes were blocked with blocking buffer for 1 h and then incubated with different primary antibodies overnight at 4 °C. After washing thrice with TBST, the membranes were incubated with anti-mouse or anti-rabbit horseradish peroxidase-conjugated secondary antibodies for 1 h at room temperature. The bands were detected by ECL reagents (Thermo Scientific) on a Bio-Rad Laboratories system.

### 4.6. Reactive Oxygen Species Measurement

After different treatments, HUVECs were loaded with CellROX Green (25 µM) in Opti-MEM (Thermo Scientific) medium and incubated for 1 h at 37 °C in a 5% CO2 culture chamber. Subsequently, HUVECs were washed thrice with PBS and digested using trypsin-EDTA solution (Thermo Scientific). ROS quantitation was carried out using the Guava Easycyte Flow Cytometer and InCyte 3.1 software (Merck Millipore, Bedford, MA, USA) based on the fluorescence intensity.

### 4.7. Enzyme-Linked Immunosorbent Assay

The concentration of C3a was measured using ELISA kits (Cusabio technology, Wuhan, China) according to the manufacturer’s protocol.

### 4.8. Detection of C5b-9 Using Cell-Based ELISA

Quantification of C5b-9 using cell-ELISA technique was performed as previously described [24]. Briefly, cell-ELISA was performed in 96-well culture plates (BD Biosciences, Franklin Lakes, NJ, USA). HUVECs were seeded at 10,000 cells/well and incubated overnight at 37 °C in 5% CO2. Cells were then cultured in media containing 10% NS at 37 °C in 5% CO_2_ for 1 h to activate the complement system. Cells were fixed with 3% paraformaldehyde. A rabbit polyclonal anti-C5b-9 antibody (Abcam, Cambridge, MA, USA) and horseradish peroxidase (HRP)-conjugated anti-rabbit IgG (Santa Cruz Biotechnology) were used as primary and secondary antibody, respectively. 3,3′,5,5′-tetramethylbenzidine (TMB, KPL, Gaithersburg, MD, USA) was added as a substrate, and the absorbance at 450 nm was measured using a microplate reader (Molecular Devices, Silicon Valley, CA, USA).

### 4.9. Flow Cytometric Analysis for C3b-Binding Cells

To detect cell surface C3b, HUVECs were treated with culture media containing 10% pooled HS before trypsinization. The cells were detached from the plate by trypsinization and incubated with primary antibodies for 30 min on ice before being washed three times with blocking solution (1% FBS in PBS) and labeled with allophycocyanin (APC)-conjugated secondary antibody (BD Biosciences, San Jose, CA, USA) for 30 min at 4 °C. Cells were analyzed using a Guava easyCyte Flow Cytometer and the InCyte 3.1 software (Merck Millipore).

### 4.10. Cell Proliferation Assay

Cell proliferation was analyzed using WST-1 cell proliferation reagent (Roche). Five thousand cells were seeded into 96-well culture plates and incubated overnight, followed by treatment with WST-1 (1:10 dilution) for 90 min at 37 °C in a cell incubator. Absorbance at 450 nm was monitored with the reference wavelength set at 650 nm using a microplate reader (Molecular Devices, San Jose, CA, USA).

### 4.11. LDH Assay

Cell death was measured using the LDH Cytotoxicity Detection Kit (Roche) according to the manufacturer’s instructions. A flat-bottom 96-well plate was used as an assay plate for each experimental plate. Triton X-100 (2 µL; 1% total volume) was added to each of the positive control wells, in addition to 100 µL of fresh medium for the negative control. A multichannel pipette was used to transfer 100 µL of supernatant from the top of each well of the experimental culture plate to the assay plate. Mixed detection kit reagent (100 µL) was then added to each of the assay wells. The assay plates were then incubated at RT in the dark for 15 min, after which they were read using a microplate reader at a reference wavelength of 490 nm (Molecular Devices).

### 4.12. Immunofluorescence Assay for the Deposition of C5b-9

Cells were seeded onto a microscopy cover glass in 24-well tissue culture plates at a density of 100,000 cells/well. After culturing overnight, the culture medium was removed and washed with PBS. The cells were fixed in 4% paraformaldehyde and blocked with 3% bovine serum albumin (BSA) in PBS. Rabbit polyclonal anti-C5b-9 (Abcam) was used as the primary antibody. Cells were incubated with a primary antibody and then incubated with Alexa Fluor-conjugated goat anti-rabbit or goat anti-mouse antibody (Invitrogen, Carlsbad, CA, USA). Nuclei were stained using 4,6-diamidino-2-phenylindole (DAPI). Cells were mounted with Vectashield^®^ (Vector Laboratories Inc., Burlingame, CA, USA) and examined under an Eclipse E400 microscope (Nikon Instruments Inc., Melville, NY, USA). Images were captured using a Nikon Digital site Fi3 and analyzed using NIS element BR (Nikon Instruments Inc.).

### 4.13. Antibody Array for Cytokine Production

Heated human serum or normal human serum was added to UPM-treated HUVECs for 1 h, followed by replacement of the medium and incubation for 24 h. The culture supernatant was collected and applied to a Proteome Profiler Human XL Cytokine Array Kit (R&D system, Minneapolis, MN, USA). The assay was performed according to the manufacturer’s instructions. Each spot signal was visualized with a Bio-Rad ChemiDoc Imaging system (Hercules), and signals were quantified with Quick Spots image analysis software (R&D systems).

### 4.14. Statistical Analysis

Results are shown as means ± standard deviations. A two-tailed Student’s *t*-test, one-way ANOVA, and two-way ANOVA were used to assess the significance of the difference between groups. Statistical significance at *p* values of <0.05 and <0.01 is indicated by * and **, respectively.

## 5. Conclusions

**Figure 7:**
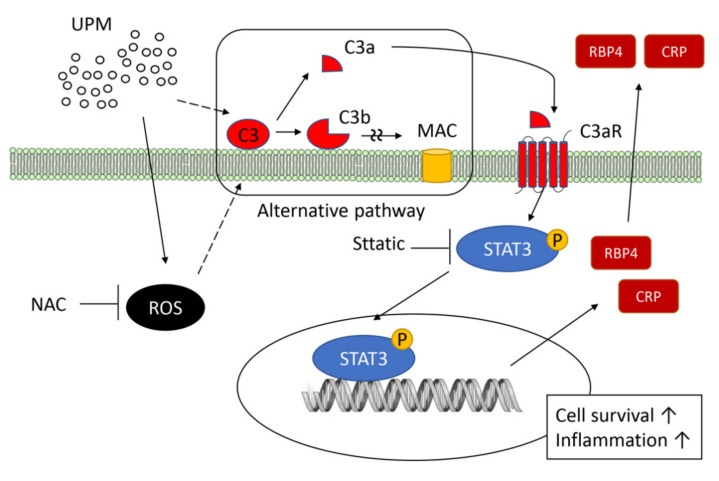
Schematic summary showing the activation of the complement system by UPM on human endothelial cells and its effects. UPM activates the complement system in human endothelial cells, in which ROS could be associated with complement activation. Dotted lines indicate unclear mechanisms in complement activation. C3a from complement activation induces the phosphorylation of STAT3, which increases cell proliferation and production of inflammatory cytokines.

This is the first study to provide direct experimental evidence that the complement system can be activated in human endothelial cells by UPM. The production of ROS in endothelial cells following UPM treatment could be associated with complement activation. At the non-toxic levels of UPM, complement activation did not induce cell death but rather enhanced the cell proliferation and production of inflammatory cytokines. A schematic summary describing the activation of the complement system by UPM on human endothelial cells and its effects is presented in Figure 7. These findings provide novel insights into the pathogenic mechanism of vascular inflammation elicited by air pollution.

## Figures and Tables

**Table 1:** List of primers used for RT-qPCR.

Gene	Sense Primer	Anti-Sense Primer
*GAPDH*	GGT ATC GTG GAA GGA CTC	GTA GAS GCA GGG ATG ATG
*CD46*	TTT GAA TGC GAT AAG GGT TT	GAG ACT GGA GGC TTG TAA
*CD55*	GGC AGT CAA TGG TCA GAT A	GGC ACT CAT ATT CCA CAA C
*CD59*	AAG AAG GAC CTG TGT AAC TT	GAG TCA CCA GCA GAA GAA
*Factor H*	TGT GGT TAC AAT GGT TGG TCT GAT	GGT AGC ACT GAA CGG AAT TAG GT
*CRP*	CAC TTC TAC ACG GAA CTG	AAT CTC ATT GTC TTG TCT CTT
*RBP4*	CCC TGC CAA GTT CAA GAT	ACG ATC CAG TGG TCA TCA

## Data Availability

Data available in a publicly accessible repository that does not issue DOIs.
